# In silico evaluation of a control system and algorithm for automated insulin infusion in the ICU setting

**DOI:** 10.1186/1475-925X-9-35

**Published:** 2010-07-20

**Authors:** José L Ortiz, Marcelo W Guarini, Gisella R Borzone, Pablo R Olmos

**Affiliations:** 1 Department of Electrical Engineering, College of Engineering, Pontificia Universidad Católica de Chile, Santiago, Chile; 2 Department of Nutrition, Diabetes & Metabolism, College of Medicine, Pontificia Universidad Católica de Chile, Santiago, Chile; 3 Department of Respiratory Diseases, College of Medicine, Pontificia Universidad Católica de Chile, Santiago, Chile

## Abstract

**Background:**

It is known that tight control of glucose in the Intensive Care Unit reduces morbidity and mortality not only in diabetic patients but also in those non-diabetics who become transiently hyperglycemic. Taking advantage of a recently marketed subcutaneous glucose sensor we designed an *Automatic Insulin Infusion System *(AIIS) for inpatient treatment, and tested its stability under simulated clinical conditions.

**Methods:**

The system included: reference glucose, glucose sensor, insulin and glucose infusion controllers and emergency infusion logic. We carried out computer simulations using Matlab/Simulink^®^, in both common and worst-case conditions.

**Results:**

The system was capable of controlling glucose levels without entering in a phase of catastrophic instability, even under severe simulated challenges. Care was taken to include in all simulations the 5-10 minute delay of the subcutaneous glucose signal when compared to the real-time serum glucose signal, a well-known characteristic of all subcutaneous glucose sensors.

**Conclusions:**

When tested *in-Silico*, a commercially available subcutaneous glucose sensor allowed the stable functioning of a proportional-derivative Automatic Insulin Infusion System, which was able to maintain glucose within acceptable limits when using a well-established glucose response model simulating a patient. Testing of the system *in vivo *using animal models is now warranted.

## Background

Diabetes Mellitus affects 5.1 per cent of the world's adult population [1], with a prevalence of up to 12.4% among hospitalized patients [2].

It has been shown that "tight control of blood glucose" (i.e. between 80 and 110 mg × dL^-1 ^= 4.4-6.1 mmol × L^-1^) in critically ill patients reduces dramatically the mortality from 8.0% to 4.6% in Intensive Care Unit (ICU) patients. Additionally, it reduces bloodstream infections by 46%, renal failure by 41%, transfusions by 50% and polyneuropathy by 44%. Interestingly, these results apply to those individuals who -being *diabetic or not*- have blood glucose on admission > 110 mg × dL^-1 ^[6.1 mmol·L^-1^] (up to 76% of ICU patients) [3-6]. Tight control of blood glucose is also useful in Coronary Care Units, where 33% of patients are diabetics and 33% have glucose intolerance. As a token of the importance of the subject, in Portland, Oregon, USA, tight control of blood glucose with intravenous insulin infusion reduced wound infection by 50%, hospital stay by 56% and mortality by 67% in diabetic patients who underwent open heart surgery [7].

**Figure 1:**
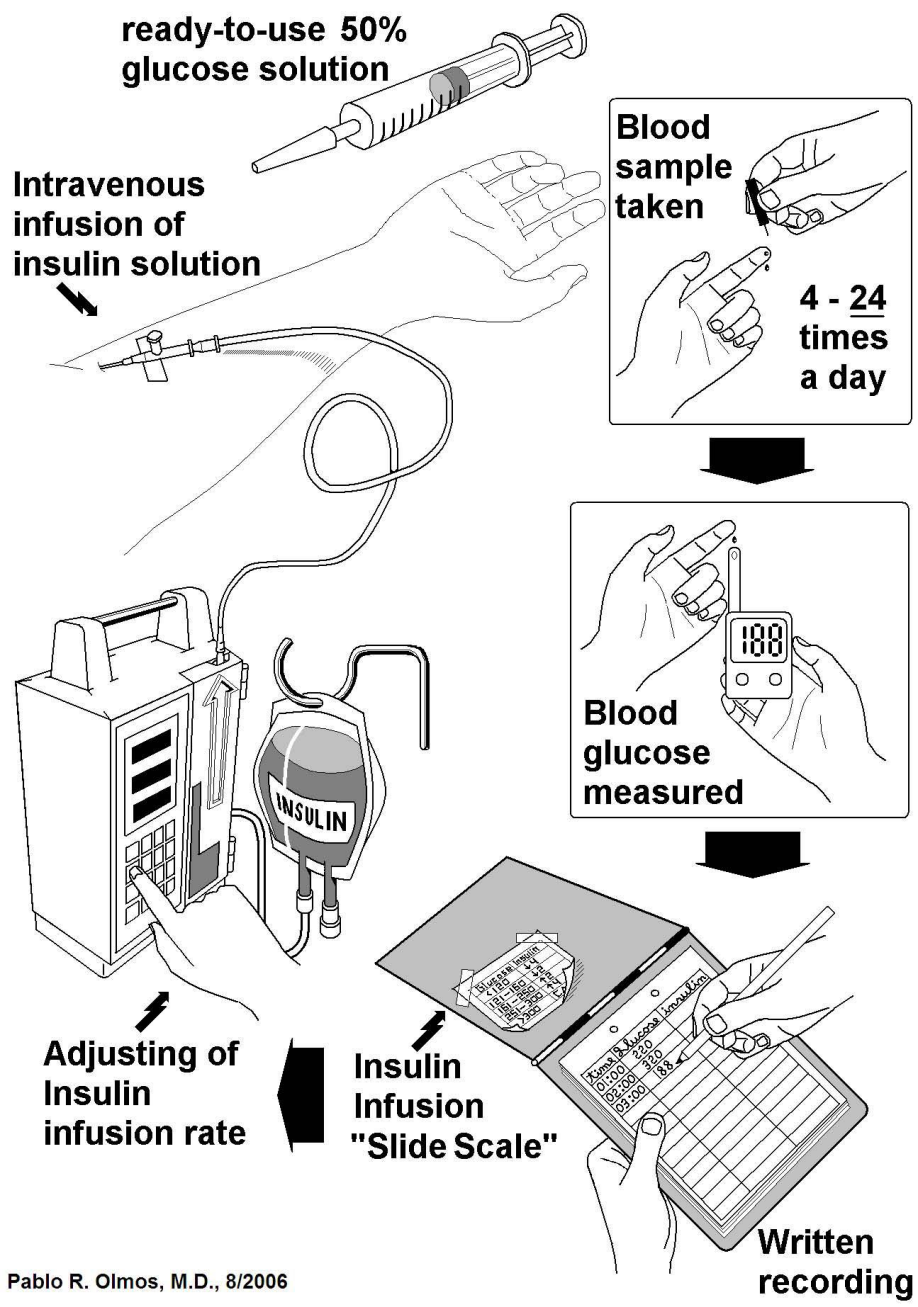
**Current in-patient management of intravenous insulin infusion**. Current in-patient management of intravenous insulin infusion(**see text**) Nurses have to measure capillary blood glucose every 1-6 hours, and after manually recording date, time and value, they read a "slide scale" previously estimated by the attending physician, that helps them to adjust the insulin infusion rate for the next period.

The current standard of care for hospitalized diabetic patients (Figure 1) requires the measurement of capillary blood glucose at least 4 times a day, and by the use of a simple algorithm ("slide scale"), adjustment of the insulin infusion rate, usually by means of an infusion pump. In cases in which "tight control" is needed, capillary blood sampling has to be done every hour, increasing personnel workload. Hypoglycemia (i.e., serum glucose < 60 mg/dl) is a frequent side effect, and must be corrected by intravenous glucose.

Thus, there is a need for automated protocols of intravenous insulin administration for hyperglycemic patients in the ICU [8,9].

In this context, we studied the behavior of an automated intravenous insulin infusion system (**AIIS**) designed by the authors, that uses an FDA-approved subcutaneous glucose sensor [10,11]. This sensor has been shown to be useful for outpatient care by enabling individuals with type-1 diabetes to manually adjust their subcutaneous insulin doses according to the glucose levels displayed by the device [11,12].

Our aim was to use Matlab/Simulink^® ^to develop a computer program for the **AIIS**, and to challenge this system repeatedly in a fashion that in some cases was similar to the worst clinical conditions possible to find in patients with diabetes in the ICU. What we considered a success was the AIIS being able to control blood glucose without becoming instable even in the worst conceivable circumstances.

Our concerns about potential instability of the AIIS originated in a characteristic of the subcutaneous glucose sensor, i.e. its 5 to 10-min. delay when compared to changes in blood glucose. We hypothesized that this delay, by altering the phase of feedback loop of the AIIS, could result in catastrophic instability. However, the results of the simulations showed that the system behaves with a high level of stability under a wide range of clinical conditions likely to be encountered in the ICU setting.

## Methods

### I. Architecture of the AIIS

**Figure 2:**
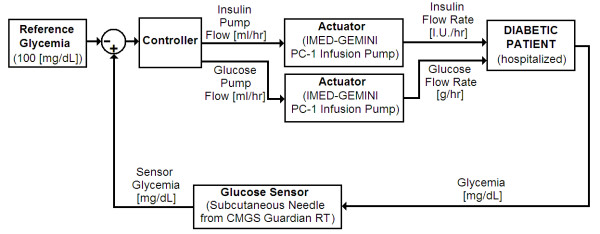
**System diagram of the "*Automatic Insulin Infusion System" *(AIIS) for Inpatient Treatment**. System diagram of the "*Automatic Insulin Infusion System" *(AIIS) for Inpatient Treatment(see text). Glucose concentration measured by the subcutaneous sensor is compared to the reference glycemia (100 mg/dL). The result becomes the input for the controller. If the result is either normoglycemia (90-120 mg/dL) or hyperglycemia (> 120 mg/dL), or if the glycemia is rising too fast, then the controller signals the insulin pump, that releases insulin solution at a particular rate (international units per hour). On the other hand, if the input to the controller reflects a hypoglycemia (i.e., glycemia < 60 mg/dL), or if the glycemia, albeit being within normal range, is falling too fast, then the controller signals the glucose pump. Every five minutes, the subcutaneous glucose sensor submits the results of a new glucose estimate to the input of the controller, and the whole process is repeated.

**Figure 3:**
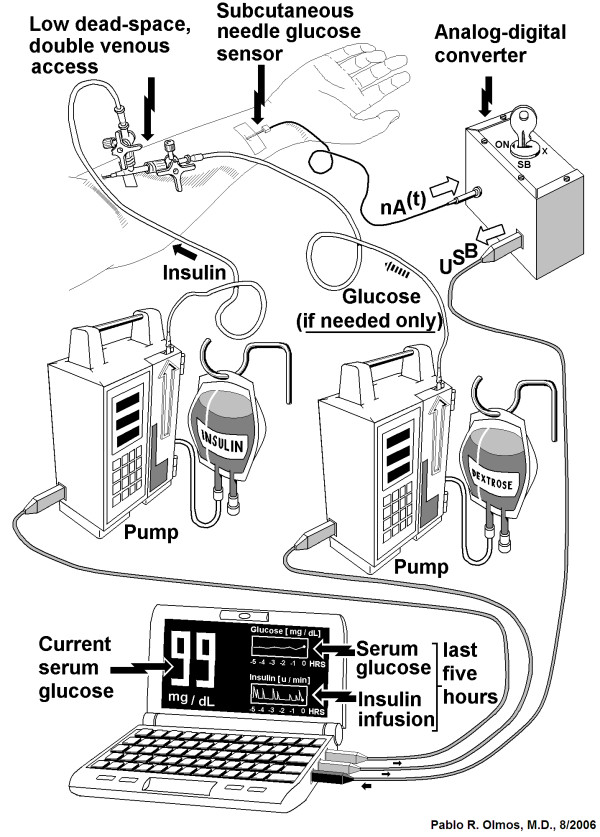
**"*Automatic Insulin Infusion System" *(AIIS) *for *Inpatient Treatment: Proposed architecture**. Proposed architecture of the "*Automatic Insulin Infusion System" *(AIIS) *for *Inpatient Treatment (see text). The nano amperimetric current produced by the subcutaneous glucose sensor located at the forearm of the patient, is shown entering an analog-digital converter. This device has two tasks. (a) To continuously measure the current from the sensor, and average it every five minutes, and (b) to transform the 5-minute average into a digital signal, that is submitted to the computer (where the controller is located) by means of an USB cable. From the computer, two USB cables are connected to infusion pumps, one for insulin, and the other for glucose solution. Note the low-dead-space catheter placed in a vein of the forearm of the patient. The low dead space allows for a rapid transition from insulin to glucose infusion and vice-versa.

Our closed-loop control system is shown in Figures 2 and 3. The glycemia of the patient acquired by the glucose sensor [13,14] is compared with a desired (reference) blood glucose level and the difference between these two values (the error) is processed by the controller, generating the signals for the actuators (insulin and glucose [dextrose in water] pumps).

#### I-A. Reference Glycemia

Normal fasting glycemia in adults varies between 70 and 99 mg × dL^-1^. In the case of an hospitalized patient, clinicians tend to keep the glycemia (***G***) below 120 mg × dL^-1^, but away from hypoglycemia. As symptoms of hypoglycemia start at ***G ***< 60 mg × dL^-1^, we set the reference level at 100 mg × dL^-1^, with an acceptable range of 90 to 120 mg × dL^-1^, near the "tight control" of blood glucose level (80-110 mg × dL^-1^) and safely away from hypoglycemia.

#### I-B Glucose Sensor

We choose the Medtronic Minimed (California, USA) Continuous Glucose Monitoring System (CGMS), a subcutaneous needle sensor capable of providing readings representative of blood glucose concentrations every 10 seconds, that are averaged every 5 minutes, totalizing 288 glucose values per day, to be either stored or displayed [11,13,14]. Its operation is based on the oxidation of glucose in the presence of oxygen and the enzyme glucose oxidase, generating an electrical current that is proportional to interstitial and blood glucose levels.

The sensor has been shown to be of value to improve outpatient control by deferred physician-supervised analysis. Its version with glucose reading display [10] allows patients to manually modify their own subcutaneous insulin dose (when using syringes) or infusion rate (when using a wearable insulin pump).

Based on the work of Chee et.al. (on the use of CGMS in critically ill patients) [15], and of Steil et.al. [16], we have developed a mathematical model of the CGMS sensor. For a sensor signal *S(t) *reacting to glucose level *G(t) *with sensitivity *α *(nano Ampères per mg/dL), the model equation becomes,(1)

In equation 1, τ is the time constant that defines an immediate proportional response, and operationally represents the time elapsed for the signal from the sensor to reach 63% of the equilibrium when compared to the intravenous glucose concentration. T_D _is a transport lag [16]. A white Gaussian noise to the sensor signal was added to our model, assuming a mean = 0.0 and a variance = 100 [17].

In their publication, Steil et. al. [16] included a graph of the simultaneous measurement of plasma and subcutaneous glucose, showing no transport lag (i.e., T_D _= 0.0) [16,18]. Of the two glucose sensors presently being developed by Medtronic Minimed, the subcutaneous glucose sensor has been shown to have a time constant ranging from 2 to 12 min. The graph by Steil et.al. depicted a time constant (τ) of 5.77 minutes [16,18], which is thought to be primarily related to interstitial glucose equilibration.

α in equation 1 is equal to 0.23 nA × [mg × dl^-1^]^ -1 ^[19]. Using data provided by the manufacturer we included lower and upper limit saturations of 40 and 400 mg × dl^-1^, respectively.

#### I-C. Controller

The controller of the AIIS can be divided into three main parts: the insulin infusion pump controller, the glucose infusion pump controller, and the emergency infusion logic (Figures 2 and 3).

The insulin infusion pump controller is based on the algorithm proposed by Marliss et.al. in 1977[20], in the form later published in 1981 by Broekhuyse et.al. [21], whose original equations were as follows,(2a)(2b)

In equation 2a, *IR *is the insulin infusion rate effectively being pumped [mU × min^ -1^], *M *is the maximum infusion rate possible [mU × min^ -1^], *S *[dL × mg^-1^] is the slope of the hyperbolic tangent curve, and *B *[mg × dL^-1^] is the blood glucose level at which half maximum infusion rate is chosen to occur (as determined from the *tanh *function curve).

In equation 2b (see "Emergency Infusion Logic", below)*, IR' *is the infusion rate in ml × hr^-1 ^as determined by the insulin infusion controller, *G*_*0*_[mg × dL^-1^] is the last available average glycemia, *G*_*crit *_is the critical serum glucose level in mg × dL^-1 ^below which intravenous glucose infusion must begin, (see also "glucose infusion controller" and equation 6, below), and *IR*_*min *_[mU × min^ -1^] is the minimum rate of insulin infusion.

In equation 2a, *DF *is the Differential Factor [mg × dL^-1^], which is computed as,(3)

where the parameters *K*_*1 *_= 1 [mg × dL^-1^]^-2 ^and *K*_*2 *_= 10 [nondimensional] [21], and the original method for calculating A [mg × dL^-1^] is as follows,(4)

where *G*_*i *_[mg × dL^-1^] is the average glycemia determined *i *minutes ago.

**Figure 4:**
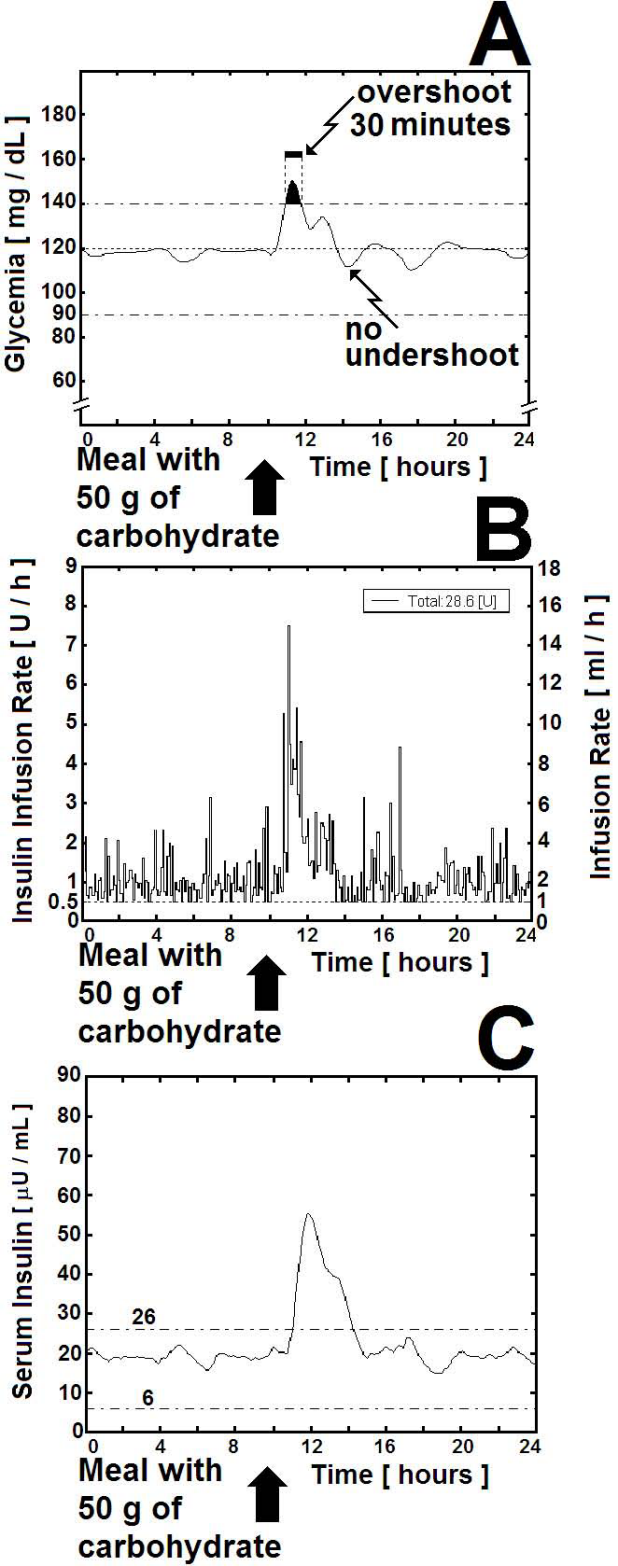
**"*Automatic Insulin Infusion System" *(AIIS) for Inpatient Treatment: Simulation of mild positive challenge**. "*Automatic Insulin Infusion System" *(AIIS) for Inpatient Treatment: Simulation of mild positive challenge given by a standard meal containing 50 grams of carbohydrate in a 70-Kg patient with Type-1 Diabetes Mellitus and average hepatic sensitivity (Sh = 0.5). See also fifth row in Table 1. (A) Glycemia [mg × dL^-1^]. The arrow shows the start of the mild positive challenge. The black area under the curve shows an overshoot that after reaching a peak at 150 mg × dL^-1^, lasts for 30 minutes. The time it takes for the blood glucose to return to a value just below 120 mg × dL^-1 ^is 90 minutes (T_S _= 90 minutes). (B) The insulin infusion rate moves around an average of 1.0 I.U. × h^-1 ^before the challenge, i.e. around 0.35 I.U. per Kilogram of body weight in a 70-Kg individual, which is the normal basal insulin secretion (see the text). After the challenge, the insulin infusion rises up to 7.0 I.U. × h^-1 ^during 90 minutes. Please observe that the insulin infusion rate is not a continuous function but a step function representing orders given by the controller to the clinical infusion pumps, whose flow rate has to be adjusted by steps not smaller than 0.1 mL × h^-1 ^(see "Actuators", part I-D in methods section, above). (C) The estimated serum insulin concentration moves around an average of 20.0 μI.U. × mL^-1 ^before the challenge, and then rises up to 55 μI.U. × mL^-1 ^for 90 minutes.

Since the CMGS System provides one measurement of glycemia every 5 minutes rather that at every minute, we propose the following modification of equation (4):(5)

where *G*_*0 *_[mg × dL^-1^] is the last reading of glycemia, and *G*_*5 *_and *G*_*10 *_are the previous readings [mg × dL^-1^] five and ten minutes earlier respectively. The coefficients accompanying the glycemia readings were obtained using as a reference the experimental data published by Broekhuyse et.al. [21] and the glycemia and insulinemia profiles from a 69.5 Kg male type 1 diabetic patient being controlled by the artificial pancreas of Albisser et.al. [22,23] which used the algorithms corresponding to the aforementioned equations (2),(3) and (4). Needing blood samples for serum glucose *every minute*, Albisser's artificial pancreas was able to function for a limited period of time. However, because it allowed a fair glycemic control, we considered adequate to emulate its profile, but using the three glycemia readings obtained every 5 minutes as shown in Equation (5). With Albisser's data and the least square approach to find the optimum parameters, we reproduced the glycemic control as shown in Figure 4, but using equation (5) instead of equation (4).

From the point of view of the control theory, considering the current glycemia in addition to the differences with previous values in equations (2), (3) and (5), allowed us to build a proportional-derivative control model, as reviewed by Parker et. al. [24].

The Glucose Infusion Pump Controller: This subsystem was included in our AIIS as an emergency measure to avoid hypoglycemia. In normal circumstances, the insulin infusion alone should be enough to keep an adequate glycemic control. This emergency subsystem is activated when serum glucose falls below 60 mg × dL^-1 ^(hypoglycemia). It has been determined that for the glucose infusion in these circumstances, proportional control (P) provides adequate performance [25]. The controller equation is given by equation 6,(6a)(6b)

where *GR *is the glucose infusion rate in ml × h^-1^, *G*_*crit *_is the critical serum glucose level in mg × dL^-1 ^below which the infusion must begin, and *G*_*0 *_is the last 5-minute-averaged glycemia reading. *K*_*G *_is the controller gain. *GR *is always positive because the controller activates itself only when *G*_*0 *_falls below *G*_*crit*_. The definition of *GR' *is given in the next paragraph, below.

Emergency Infusion Logic: It has been included in our system to both shut down the insulin infusion to a minimum and activate the glucose infusion whenever a critically low glycemia (*G*_*crit*_) is detected. Once the glycemia returns to a value that is above the critical level, insulin infusion returns back to normal operation and glucose infusion is disabled. The activation or deactivation of each of the infusion pumps were summarized in equation 2b for insulin (see above) and in equation 6b for glucose, where *GR' *is the infusion rate in ml × hr^-1 ^as determined by the glucose infusion controller, and *GR *is the infusion rate effectively leaving the glucose pump.

The insulin infusion has a minimum value that is different from zero (i.e. *IR*_*min*_≠ 0; see Equation 2, above) for two reasons. First, considering that the insulin and glucose infusions take place through a catheter inserted in a vein, it is essential to ensure a minimum permanent flow to avoid the occlusion of the catheter (KVO, "keep vein open" feature). Second, in diabetic patients lacking endogenous production of insulin (Type-1 diabetes mellitus), it is essential to ensure a minimal serum concentration of insulin in order to avoid a massive lipolysis with ensuing ketoacidosis.

#### I-D Actuators

As described above, the AIIS has two infusion mechanisms regulating blood glucose concentration: insulin infusion and glucose infusion using the IMED Gemini PC-1 infusion pump produced by Alaris Medical Systems (San Diego, CA, U.S.A.). This pump fulfills the specifications of our system in terms of range of infusion rate, computer communication capabilities and accuracy. The ideal operation of the device for the simulations considered a minimum infusion rate of 1.0 ml × h^-1^, a maximum rate of 500 ml × h^-1^, and rate steps of 0.1 ml × h^-1 ^[26]. Although the IMED Gemini PC-2 model could have been more convenient due to its double channel capacity that allows the infusion of two solutions simultaneously and independently, for simplicity we used two separate IMED Gemini PC-1 pumps. (Figure 3).

#### I-E Diabetic Patient

In order to carry out the computer simulations aimed to test the behavior of our system, we used a mathematical model of human glucose physiology based on the work by Lehmann, Deutsch & Hermanyi [27,28], which assumes a patient completely lacking endogenous insulin secretion. It contains a single compartment of extracellular glucose, where it enters via both intestinal absorption and hepatic glucose production. Similarly, the gastric emptying rate that provides the glucose flux from the stomach to the small intestine is controlled by a process defined as a function of the carbohydrate content of the ingested food. The glucose is removed from the extracellular space by insulin-independent utilization in the central nervous system and red blood cells, and also by the insulin-dependent utilization by the liver and peripheral tissues (muscle and fat). Additionally, glucose excretion is considered to take place through the kidneys when the glycemia exceeds renal glucose threshold.

The model accounts separately for the glucose entering the peripheral tissues and the glucose entering/leaving the liver, an approach that allows to assign a range of values to patient-specific parameters, such as hepatic insulin sensitivity [26,27]. In this context, the model summarizes the glucose processing by the liver as the *net hepatic glucose balance *(NHGB), which is the algebraic sum of the glucose produced by both *neoglucogenesis *and glycogen breakdown, minus the glucose removed by glycogen synthesis. The NHGB is a function of both the glucose and the insulin plasma concentrations (see below) [29].

In fact, the Lehman & Deutsch model is based on four equations. The first one is a differential equation describing the change in plasma insulin concentration (dI/dt). The second is also a differential equation that now describes the build-up and deactivation of the active insulin pool (dI_a_/dt). The third and fourth equations describe the rate of insulin absorption from the subcutaneous tissue. As our AIIS injects insulin directly into the bloodstream, we did not use these last two equations. On the other hand, the overall rate of peripheral plus insulin-dependent glucose utilization (G_out _, mg × Kg^-1 ^× h^-1^) depends on an equation whose two main variables are the current glucose level (G, mg × dL^-1^) and the insulin concentration (I*_eq _, mU.I. × L^-1^). In the same equation, the most important constant is the slope of peripheral glucose utilization versus insulin line (c, 0.045 mg × min^-1 ^× mIU^-1 ^× L).

When the patient eats carbohydrates, the Lehman & Deutsch model allows for calculation of the rate of change of glucose in the gut (G_gut _, mg × dL^-1^), which depends on the glucose load and the rate of gastric emptying. This G_gut _in turn, allows the continuous calculation of the systemic appearance of glucose via absorption from the gut (G_in _, mg × dL^-1 ^× h^-1^).

The NHGB (mg × h^-1^), which can be either positive or negative, depends on effective plasma insulin, that is, plasma insulin (I*_eq _, mU.I. × L^-1^), times the patient-specific hepatic insulin sensitivity (Sh), an adimensional number that has a normalized value between 0 and 1. Published tables [27,29] allow for the calculation of NHGB for any effective insulin level.

Finally, the change of glycemia with time (dG/dt, mg × dL^-1 ^× h^-1 ^) is given by a differential equation depending on G_in_(t), plus NHGB(t), minus G_out_(t), and minus G_ren_(t). Where G_ren _is the rate of renal excretion of glucose (mg × h^-1^).

The main advantage of the Lehman & Deutsch model resides in its explicit anatomical compartments, taking into account the function of the body organs involved. For the same reason, it is easy to introduce modifications in the model, as it was necessary with our own simulations. The Lehman & Deutsch model was validated clinically in 1994 by their own authors [27].

More recently, others [30] have tried to improve the understanding of the glucose-insulin interactions by means of an approach based in 4 unit processes, i.e., liver, gastrointestinal tract, muscle & adipose tissue and beta cell. In our view however, this interesting new model underestimates the endogenous glucose production [31,32].

### II.- Simulations of the Behavior of the AIIS in Hospitalized Patients

To test the behavior of the AIIS in normal and in the worst possible scenarios, we designed the series of cases summarized in tables 1 and 2. Simulations were carried out in Matlab/Simulink^® ^v6.5 from Mathworks Inc., using a Pentium IV 1,800 MHz computer, equipped with 256 MB RAM. Under these conditions, each 24-hour simulation took about 15 seconds to complete.

**Table 1:** Positive challenges.

			**Overshoot (Glycemia > 140 mg × dL**^**-1**^**)**			**Undershoot (glycemia < 60 mg × dL**^**-1**^**)**		
**Type of Challenge**	**Weight (Kg)**	**Hepatic Sensitivity (Sh)**	**Peak (mg × dL**^**-1**^**)**	**Duration (min)**	**T**_S_* (min)	**Peak (mg × dL**^**-1**^**)**	**Duration (min)**	**T**_S_* (min)

		0.7	144	38	72	no	no	n/a
		
	80	0.5	154	72	95	no	no	n/a
		
		0.3	168	102	144	no	no	n/a
	
		0.7	144	30	90	no	no	n/a
		
Mild ¶	70	**0.5**	**150**	**30**	**90**	**no**	**no**	**n/a**
		
		0.3	163	84	135	no	no	n/a
	
		0.7	143	22	42	no	no	n/a
		
	60	0.5	150	46	66	no	no	n/a
		
		0.3	159	72	108	no	no	n/a

		0.7	164	54	72	no	no	n/a
		
	80	0.5	172	76	92	no	no	n/a
		
		0.3	182	86	108	no	no	n/a
	
		0.7	163	50	70	no	no	n/a
		
Moderate §	70	0.5	170	30	60	no	no	n/a
		
		0.3	181	78	96	no	no	n/a
	
		0.7	162	50	70	no	no	n/a
		
	60	0.5	170	58	72	no	no	n/a
		
		0.3	183	72	84	no	no	n/a

		0.7	170	30	31	no	no	n/a
		
	80	0.5	176	36	37	no	no	n/a
		
		0.3	177	41	42	no	no	n/a
	
		0.7	175	30	55	no	no	n/a
		
Severe †	**70**	**0.5**	**180**	**15**	**120**	**no**	**no**	**n/a**
		
		0.3	191	36	39	no	no	n/a
	
		0.7	189	30	31	no	no	n/a
		
	60	0.5	188	29	30	no	no	n/a
		
		0.3	201	36	38	no	no	n/a

**Figure 5:**
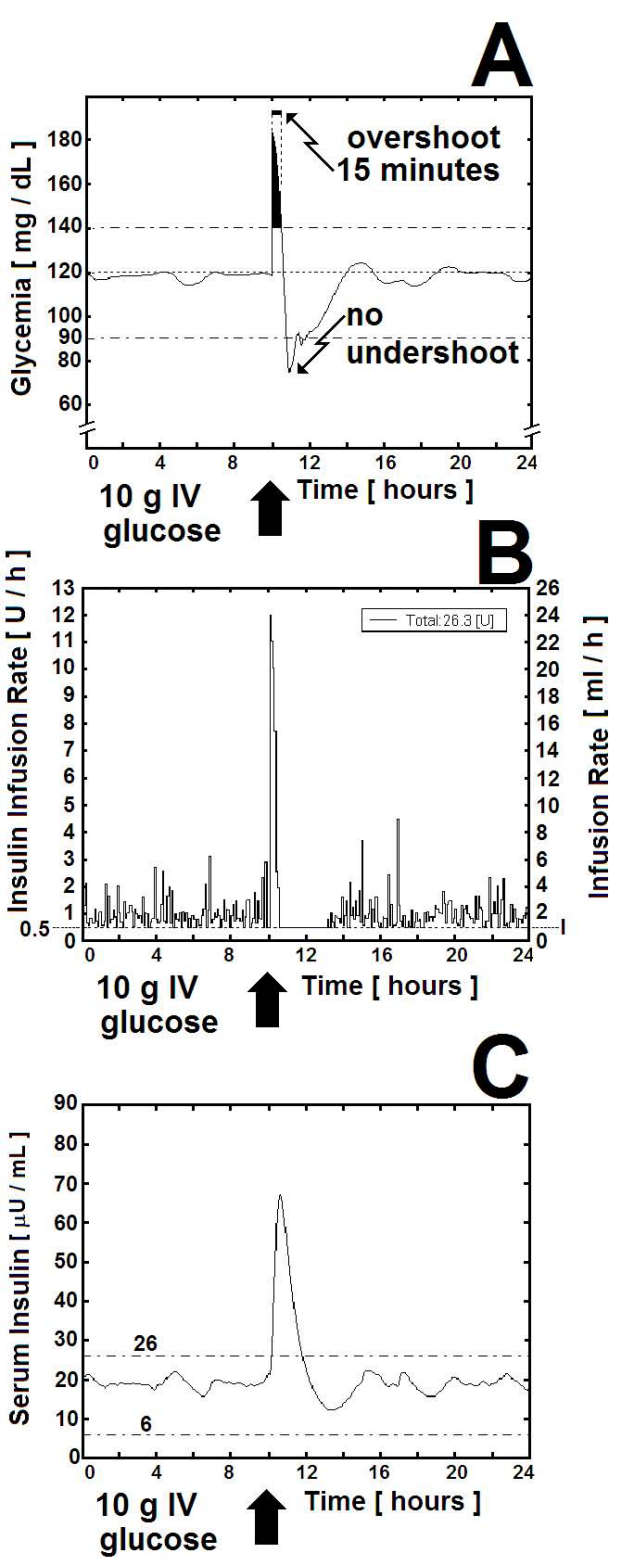
**"*Automatic Insulin Infusion System" *(AIIS) for Inpatient Treatment: Severe positive challenge**. "*Automatic Insulin Infusion System" *(AIIS) for Inpatient Treatment: Severe positive challenge given by the sudden intravenous injection of 10 grams of glucose into a 70-Kg patient with Type-1 diabetes mellitus and average hepatic sensitivity (Sh = 0.5) as shown in the 23^d ^row of Table 1. (A) Glycemia [mg × dL^-1^]. The arrow shows the start of the severe positive challenge. The black area under the curve is the overshoot that lasts for 15 minutes. Although the time it takes for the blood glucose to return to a value just below 120 mg × dL^-1 ^is 16 minutes, the real T_S _= 120 minutes, which is the time it takes for the system to stabilize itself within the 90-120 mg × dL^-1 ^range. There is no undershoot, as the glycemia does not fall below 60 mg × dL^-1^. (B) As in the case of figure 4-B, the insulin infusion rate moves around an average of 1.0 I.U. × h^-1 ^before the challenge. Immediately after the challenge, the insulin infusion rises up to 12.0 I.U. × h^-1 ^during 15 minutes. This was followed by minimal infusion rate for the next 45 minutes (KVO for the Abbott pump) in order to avoid excessive hyperinsulinemia (see 5-C, below). Please observe that the insulin infusion rate is not a continuous function but a step function representing orders given by the controller to the clinical infusion pumps, whose flow rate has to be adjusted by steps not smaller than 0.1 mL × h^-1 ^(see "Actuators", part I-D in methods section, above). (C) The AIIS response to the severe positive challenge produced a period of hyperinsulinemia that lasted for 55 minutes. The elevated levels of serum insulin were the result of the sharp increase in insulin infusion (as shown in 5-B, above), and its duration was limited to 55 minutes thanks to the 45-minute period of minimal insulin infusion that ensued. The result was a undershoot-free normalization of glycemia.

Note the inter-individual variations with body weight and/or hepatic sensitivity changes.

(**bold**) The two rows in **bold numbers **are the ones represented in Figures 4 and 5 respectively

(*) T_S _= settling time, minutes for the glycemia to return to the 90-120 mg × dL^-1 ^range

(¶) Mild challenge = 50 g of oral carbohydrate (from 50 g of starch)

(§) 50 g of oral glucose (250 g of grapes)

(†) 10 g intravenous glucose (20 mL of 50% dextrose in water) in 30 seconds

**Table 2:** Negative challenge in 70-Kg type-1 diabetics.

		**Undershoot (glycemia < 60 mg × dL**^**-1**^**)**			**Overshoot (Glycemia > 140 mg × dL**^**-1**^**)**		
**Challenge**	**Hepatic Sensitivity (Sh)**	**Peak (mg × dL**^**-1**^**)**	**Duration (min)**	**T**_S_* (min)	**Peak (mg × dL**^**-1**^**)**	**Duration (min)**	**T**_S_* (min)

10 units of regular insulin administered intravenously in 2 seconds	0.7	10	2	22	no	no	n/a
	
	0.5	12	5	60	no	no	n/a
	
	0.3	31	7	45	no	no	n/a

Note the inter-individual variations with hepatic sensitivity changes.

(*) T_S _= settling time, minutes after the start of the challenge for the glycemia to return to the 90-120 mg × dL^-1 ^range

## Results

### I Positive Challenges

Table 1 shows the positive challenges that we expected to be controlled by the AIIS, i.e. all perturbations tending to increase glycemia. We assumed that a single perturbation occurred at the hour 10:00 within the 24-hour simulation span.

#### I-A Mild Challenge

The mild challenge simulates a patient eating normally an average meal, containing 50 grams of complex carbohydrates (starch), the digestion of which allows the glucose to enter the bloodstream in a slow fashion. As shown in Table 1 and in Figure 4-A, in the case of a 70-Kg diabetic with average hepatic sensitivity (Sh = 0.5), the glycemia rises temporarily to a level close to 150 mg × dL^-1^, comparable to the transient elevation of serum glucose after a meal in a normal adult. As soon as the AIIS detects this rise in glycemia, the insulin infusion rate rapidly increases in concordance with the derivative component of the control algorithm (Figure 4-B). The same algorithm makes insulin infusion rate to decrease sharply as soon as the first derivative of the glycemia reaches the positive-to-negative critical point, without falling below 1 ml × h^-1^, thus avoiding the risk of ketoacidosis (a complication that might arise if plasma insulin concentration was allowed to fall below 0.5 μU × ml^-1^). In fact, as shown in Figure 4-C, plasma insulin never falls below the lower limit of the normal fasting range (6-26 μU × ml^-1^) [33]. Furthermore, the aforementioned minimal infusion rate of 1 ml/hr corresponds to the parameter "K.V.O." of the IMED Gemini PC-1 infusion pump. The lack of an undershoot of glycemia after this mild challenge precluded the glucose infusion to start.

#### I-B Moderate Challenge

A moderate positive demand was given by the ingestion of 50 grams of glucose in the form of 250 grams of grapes. As seen in the 14^th ^row of Table 1, in the case of a 70-Kg diabetic with average hepatic sensitivity (Sh = 0.5) the glycemia rose rapidly up to a value close to 170 mg × dL^-1^, and a peak duration of 30 min. The hyperglycemia was then controlled by the AIIS by means of a sharp and sustained increase in the insulin infusion rate, that corrected glycemia after a T_S _of 60 minutes. As with the mild challenge, only the insulin infusion was required whereas the glucose pump remained inactive.

#### I-C Severe Challenge

For the severe positive challenge we selected the most abrupt method of administering glucose to a patient: injecting 20 ml of 50% dextrose-in water into the subclavian vein in 30 seconds. As seen both in the 23^d ^row of Table 1 and in Figure 5, in the case of a 70-Kg diabetic with average hepatic sensitivity (Sh = 0.5), a very steep rise in glycemia ensued after glucose injection (Figure 5-A). The fast rise in the insulin infusion rate that was ordered by the AIIS controller (Figure 5-B) was able to keep the hyperglycemic overshoot within tolerable limits, i.e., 180 mg × dL^-1 ^for 15 minutes. For some minutes after the end of the sharp insulin response, serum insulin remained high (Figure 5-C), resulting in a reduction in serum glucose to levels below 90 mg × dL^-1^, but without reaching a true undershoot value thanks to (a) a 45-minute period of minimum insulin infusion(KVO), and (b) the activation of the glucose pump. After a T_S _of 120 minutes, serum glucose returned to the 90-120 mg × dL^-1 ^range.

#### I-D Inter-Individual Variation In The Performance of the AIIS

In the year 1994, when the Lehman & Deutsch model was validated clinically, two parameters, Sh y Sp, were introduced in order to reduce the differences between observed and predicted blood glucose. Sh y Sp are, respectively, hepatic and peripheral sensitivities to insulin. Both are adimensional numbers ranging from 0.0 to 1.0.

In order to explore the inter-individual variation in the behavior of the AIIS, we challenged our own model for patients with body weight above and below 70 kg, and hepatic sensitivity (S_h _) above and below 0.5 (Table 1). For the mild positive challenge, the overshoot varied between 143 and 168 mg × dL^-1^, lasting for 22-102 min., with settling times between 42 and 144 min. For the moderate positive challenges, the overshoot varied between 162 and 183 mg × dL^-1^, lasting for 30-86 min., with settling times between 60 and 108 min.

For the severe positive challenges, these figures were: overshoot of 170-201 mg × dL^-1 ^, lasting 29-41 min., with a settling time of 30-120 min. There were no "bounce back" undershoots.

### II Negative Challenges

Table 2 shows the negative challenge (a perturbation that tends to decrease glycemia) that we expected to be controlled by the AIIS. We assumed that this occurred at the hour 10:00 as a single perturbation within the 24-hour simulation span. The strong negative challenge was the intravenous injection of 10 units of regular insulin. To put this in mathematical terms, we applied the two-compartment model for intravenous insulin bolus described by Kobayashi et al. [34], using the parameters shown in their Appendix A. After injection, serum insulin concentration rose to 3,300 μU × ml^-1 ^, making the glycemia to fall below 60 mg × dL^-1^. However, once more the controller of the AIIS responded instantly, both by almost stopping insulin infusion (down to "KVO") and aggressively activating glucose infusion. As a result, the undershoot (glycemia < 60 mg × dL^-1^) was corrected after 5 min., with a Ts of 90 minutes for it to return to the 90-120 mg × dL^-1 ^range.

In Table 2, in the same 70-Kg patient having hepatic sensitivities (S_h _) of 0.7 and 0.3, the negative challenge produced glycemias of 10 and 31 mg × dL^-1 ^respectively, that lasted 2 and 7 min., with settling times of 22 and 45 min.

## Discussion

A quarter of a century ago, the introduction of capillary blood glucose monitors allowed Type-1 diabetics to *intermittently *measure their glycemia and adjust their subcutaneous insulin dosage 3 to 4 times a day using empirical algorithms. This so-called "Intensified Insulin Therapy", currently the standard of care for outpatients and an example of "open-loop glucose control", has resulted in a significant reduction in chronic microvascular complications in diabetic patients [35,36]. Continuous glucose monitoring became possible in 1999 by means of the Continuous Glucose Monitoring System (Medtronic Minimed CGMS, Northridge, CA, USA) [10,19]. In the last 5 years, the subcutaneous glucose monitoring systems using microdialysis (GlucoDay, A. Menarini Diagnostics, Italy) [37] and an improved version of the Medtronic Minimed CGMS (Guardian^® ^RT) were introduced allowing real-time glucose readings [11]. In the year 2006, R. Hovorka [38] summarized 15 clinical studies of closed-loop control in Type 1 diabetics carried out between 1960 and 2004, of which at least nine used subcutaneous sensors. These studies were oriented to *outpatient *Type-1 diabetics; with insulin being administered either subcutaneously or intraperitoneally according to empirical, semi-automatic and automatic algorithms.

The reader might argue why we have concentrated ourselves on intravenous insulin infusion in hospitalized patients (IVII-H), instead of modeling a system of continuous subcutaneous insulin infusion (CSII-H).

Although CSII has been used in outpatients as well as in hospitalized patients, its use is not advisable in patients requiring Intensive & Intermediate Care, due to the impredictable absorption of insulin from the subcutaneous tissue due to vasoconstriction. The Intravenous Insulin Infusion, on the contrary, allows for 100 per cent bioavailability of insulin even in patients with decreased perfusion of subcutaneous tissues.

In the methods section, we mentioned the use of the Lehman & Deutsch [26,27] model, in our view a valid choice, considering that some more recent approaches to glucose metabolism tend to underestimate endogenous glucose production at zero insulin [30-32].

As it was mentioned in the introduction, our aim was to explore the closed-loop glucose control under conditions observed in ICU patients. The AIIS included a subcutaneous sensor with the characteristics of the Guardian^® ^RT (Medtronic Minimed) and two infusion pumps for the intravenous administration of regular insulin or glucose. Our "*Automatic Insulin Infusion System*" proved to be stable even when subjected to the worst conceivable challenges in the clinical setting.

Our main concern when modeling our AIIS was the possibility of catastrophic instability due to the 5-10 min. delay of the subcutaneous glucose sensor. From the point of view of the control theory, it is well known that an important source of instability is precisely the delay in the feedback subsystem. The fact that the AIIS did not become instable even with the worst worst-case challenges, encourages our team to take the next step, i.e., to build a prototype for animal testing.

With respect to the negative challenge, we observed that, the higher the insulin sensitivity, the deepest the glycemia undershoot was, being compensated by a shorter hypoglycemia, and shorter settling times.

In the future, other protocols, perhaps a "Model Predictive Control" (MPC) might be used for inpatient automatic insulin infusion systems, as it has been already proposed for both intravenous [24] and subcutaneous [35,39] closed-loop insulin infusion systems. In fact, MPC for subcutaneous insulin infusion has already been tested by Schaller et. al. [40], who used either intravenous or subcutaneous (simulated by a 30-minute delay) glucose measurements. As we have used only intravenous insulin infusion in our *in-Silico *model of the AIIS, the MPC system of Schaller *et.al*. will need to be adapted for intravenous insulin when used in hospitalized patients.

We are aware that the subcutaneous glucose sensors that constitute the essential components of all continuous glucose monitoring devices do not perfectly reflect changes in blood glucose. In fact, during euglycemia, the mean absolute relative differences (MARD's) have been proven by others to be, respectively, 15.2, 21.2, 15.3 and 15.6 per cent, for the four currently available CGMs, i.e., Guardian (Medtronic, Northridge, CA, USA), Dexcom (DexCom, San Diego, CA, USA), Navigator (Abbott Diabetes Care, Alameda, CA, USA), and Glucoday (A. Menarini Diagnostics, Florence, Italy). Of these, the first three are subcutaneous needle-type, and the last one uses a microdyalysis system [41]. However the needle-type system that we decided to include in our model (the same as the one used in Guardian^®^) has an accuracy that is not significantly different from the others [41], and constitute now the best available choice.

Comparison of the performance of the AIIS to other existing solutions can be done at three different levels:

In the first level, the averaged results of 1583 ICU patients in 12 "expert-based control studies" reviewed by Moijering S., et.al. [42] were compared with the results of the AIIS. These studies used sliding scale or titration-based protocols developed on the basis of previous clinical experience [43]. Their reported mean blood glucose of 7.92 ± 1.32 mmol × L^-1 ^(142.6 ± 23.7 mg × dL^-1^) was obtained with capillary sampling every 1.44 - 3.0 hours. On the other hand, our proposed AIIS was aimed to a lower blood glucose of 100 mg × dL^-1 ^(90 to 120 mg × dL^-1^), was not dependent on repeated blood sampling, and had a more rigorous criteria for hypoglycemia (i.e., < 60 mg × dL^-1^).

At the second comparison level are the "model-based methods". In the year 2008, Chase et.al. [43] published the results of their SPRINT (Specialized Relative INsulin and Nutrition Tables), that regulated insulin administration by being fed data (both current and during the previous 1-2 hours) from 3 sources, i.e., (a) capillary glucose (every 90 minutes); (b) intravenous boluses of insulin over 15-30 seconds, and (c) enteral nutrition by nasogastric tube (not exceeding 90 mL × hour^-1^). The SPRINT has the merit of having been clinically tested [43,44], albeit with an average of 16 capillary samples per day. However, the SPRINT has been designed for tube-fed patients only.

The third comparison level is the "artificial pancreas", of which there is only one in current clinical use - the Japanese STG-22. This device is composed of three main parts: the sensor system, a computer and an operation system capable of delivering intravenous solutions of insulin or glucose. The sensor system obtains blood samples every 5 minutes by means of an indwelling double-lumen catheter, that conducts the blood towards a glucose-oxidase sensor. The glucose data is fed into the computer, programmed with a proportional-derivative algorithm whose output controls the delivery of insulin or glucose to the patient. Since the year 1983, the STG-22 has been used in more that 14,000 patients, including clinical and experimental applications [45]. The single and most transcendental difference with our AIIS is the glucose sensing system, as our device, instead of relying on repeated blood sampling, uses a much less invasive subcutaneous glucose sensor.

## Conclusions

The fact that after severely challenging the AIIS it did not become instable, even in the context of using a subcutaneous glucose sensor with a 5 to 10 min. delay, means to us that a prototype can be constructed for animal testing, under the expectations of using it in the ICU setting in order to achieve tight control of blood glucose without frequent blood sampling.

**Figure 6:**
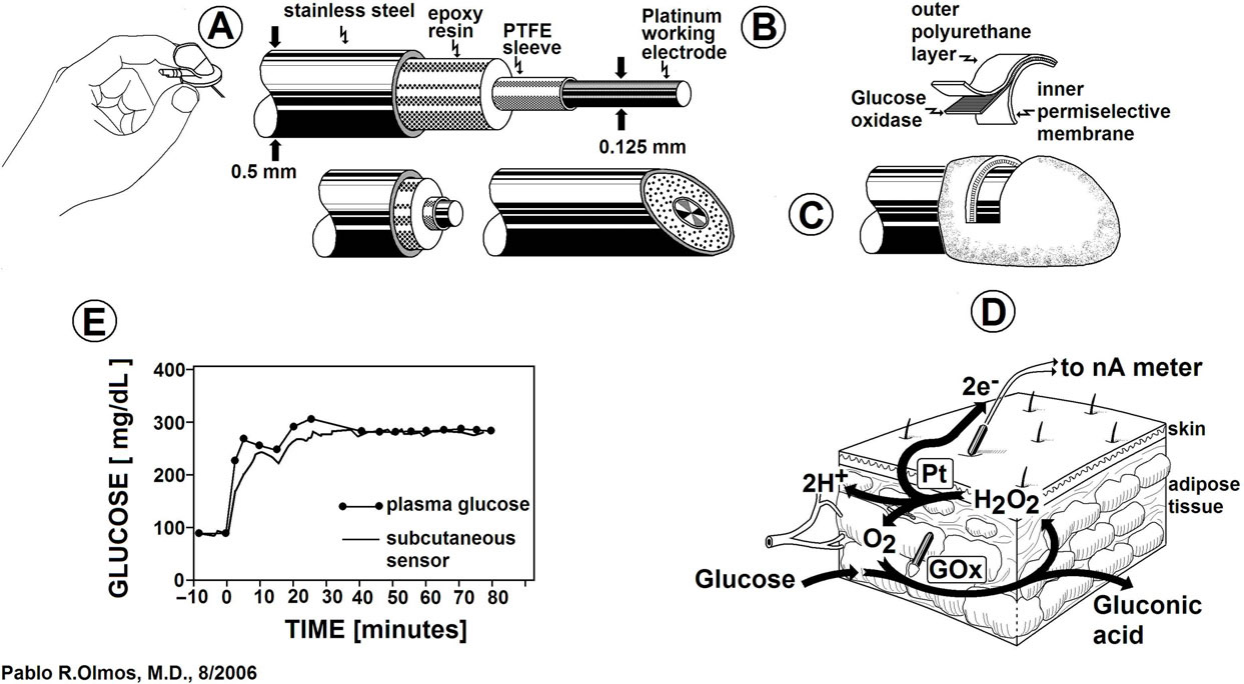
**A commercially available subcutaneous glucose sensor. Five views (A, B, C, D and E)**. (A) A commercially available subcutaneous glucose sensor. (B) Exploded-view of a *generic *glucose sensor structure. (C) The tip of the glucose sensor with its three layers. (D) The glucose sensor needle placed under the skin in the subcutaneous tissue, where the electrochemical reaction produces a DC current (nano-Amperes) that is directly proportional to the glucose concentration. (E) Response of the Medtronic Minimed subcutaneous sensor (thin line) to a step change in serum glucose (black circles) (re-drawn from data in Ref. [16])

Indeed, we believe that the closed-loop control, with subcutaneous glucose sensing (Figure 6) and intravenous insulin administration constitutes a promising approach for developing new devices oriented to the tight control of glycemia in hospitalized patients, particularly in the intensive care unit. This approach has two advantages. First, it provides a new tool to reduce mortality and morbidity in the ICU. Last, but not least, this system shall be tested in an environment with highly trained personnel and 24-hour surveillance, so the experience thus gained might one day extend its benefits to the outpatient setting, i.e., to millions of insulin-requiring diabetics worldwide.

## List of Abbreviations used

α: (greek letter alpha) Sensitivity (nA × [mg × dl^-1^]^ -1^) [Eq. 1]; A, Differential Parameter (mg × dL^-1^) [Eq. 4 and 5]; AIIS: Automatic Insulin Infusion System (for inpatient treatment); B: Blood glucose level (mg × dL^-1^) at which half-maximum infusion rate is chosen to occur [Eq. 2a]; CGMS: Continuous Glucose Monitoring System; DF: Differential Factor (mg × dL^-1^) [Eq. 2a and 3]; FDA: Food and Drug Administration (USA); G: Glycemia (blood glucose concentration, mg × dL^-1^) [Eq. 1]; G_0_: Last 5-minute averaged glycemia (mg × dL^-1^) [Eq. 5 and 6]; G_crit_: Critical glycemia (mg × dL^-1^) below which the glucose infusion controller activates itself [Eq.2b and 6b]; G_i_: Glycemia (mg × dL^-1^) determined "i" minutes ago [Eq. 4 and 5]; GFR: Glomerular Filtration Rate (mL × min^-1^), a measure of renal function; GLUT-4: Glucose Transporter - 4 (present in muscular and adipose tissues); GR: Glucose infusion rate (mL × h^-1^) [Eqs. 6a and 6b]. Note that it is in milliliters per hour (and not in mL × min^-1^) as the commercially available pumps are graduated in this way; GR': Infusion rate in mL × h^-1 ^as determined by the glucose infusion controller [Eq. 6b]; ICU: Intensive Care Unit; IR: Insulin Infusion Rate (mIU × min^-1^) [Eq.2]; IR': Insulin infusion rate (mL × h^-1^) determined by the insulin infusion controller; K_1_: = 1 [mg × dL^-1^]^-2 ^, a parameter [Eq. 3] [21]; K_2_: = 10 [adimensional], a parameter [Eq.3] [21]; K_G_: Controller gain ([mL × h^-1^] × [mg × dL^-1^]^-1^) [Eq. 6a]; KVO: "Keep Vein Open". Minimal infusion rate (= 1.0 mL × h^-1^) needed to keep venous access from being occluded by thrombosis; M: Maximal possible insulin infusion rate (mIU × min^-1^) [Eq. 2]; NHGB: Net Hepatic Glucose Balance (mg × min^-1^); SGM: Subcutaneous Glucose Monitor; S: Slope (dL × mg^-1^) of the hyperbolic tangent curve [Eq. 2]; S_P_: Peripheral sensitivity to insulin (adimensional; 0.0 ≤ S_P _≤ 1.0); S_h_: Hepatic sensitivity to insulin (adimensional; 0.0 ≤ S_h _≤ 1.0); τ: (greek letter *tau*). Time constant (minutes) [Eq. 1]; T_D_: Transport lag (minutes) [Eq. 1]

## Competing interests

The authors declare that they have no competing interests.

## Authors' contributions

JO made his MSc Thesis on Electronic Engineering on this subject. MG was adviser to JO in his thesis, contributed to write and reviewed the manuscript. GB contributed to write and critically reviewed the manuscript. PO was adviser to JO in his thesis, contributed to write the manuscript and drew the illustrations. All authors have read and approved the final manuscript.

## Author's information

JO is a BscEE (2006) and MscEE (2008)

MG is a Full Professor of Electrical Engineering, with a PhD in Electrical Engineering (University of Arizona, U.S.A. 1989)

GB is Associate Professor of Medicine (Department of Respiratory Diseases), Respiratory Diseases Specialist (The Ohio State University College of Medicine, USA, 1993), and has a PhD in Medical Biochemistry (The Ohio State University Graduate School, USA, 1993)

PO is Associate Professor of Medicine (Department of Diabetes, Nutrition & Metabolism), Endocrinologist (The Ohio State University College of Medicine, USA, 1993) and has a MSc in Biomedical Engineering (The Ohio State University Graduate School, USA, 1993)
